# Unrecognized Primary Hypothyroidism As a Possible Cause of Hyperreactio Luteinalis

**DOI:** 10.7759/cureus.13573

**Published:** 2021-02-26

**Authors:** Yash V Chauhan, Pradip P Dalwadi, Jugal V Gada, Premlata K Varthakavi, Nikhil Bhagwat

**Affiliations:** 1 Endocrinology, Topiwala National Medical College & Bai Yamunabai Laxman Nair Charitable Hospital, Mumbai, IND

**Keywords:** hyperreactio luteinalis, hypothyroidism, pregnancy, hcg, tsh, fsh, spoke-wheel

## Abstract

Hyperreactio luteinalis (HRL) is characterised by benign enlargement of ovaries in pregnancy associated with hyperandrogenism. A 19-year-old primigravida presented with breathlessness, abdominal distension and vomiting in the thirteenth week of gestation. Abdominal examination revealed distension of abdomen disproportionate to the gestational age. Ultrasound was suggestive of bilaterally enlarged multicystic ovaries with a characteristic “spoke-wheel” pattern and a diagnosis of HRL was made. Laboratory investigations revealed primary hypothyroidism and elevated testosterone. She was initiated on levothyroxine therapy. Her respiratory distress worsened on the third day of admission for which she underwent emergency laparotomy with cyst aspiration. Thyroid function tests normalized within six weeks after the initiation of therapy and remained normal for the remainder of pregnancy. Serum testosterone levels returned to normal six weeks postpartum. The elevated thyroid-stimulating hormone levels could have contributed to development of HRL by cross-reacting with human chorionic gonadotropin and follicle-stimulating hormone receptors. Hyperandrogenism and ovarian enlargement regresses with levothyroxine therapy.

## Introduction

Hyperreactio luteinalis (HRL) is considered an extreme variant of theca lutein cysts and should be suspected when a pregnant patient presents with ovarian enlargement and hyperandrogenism. It is believed to form due to β-human chorionic gonadotropin (β-HCG) stimulation, either due to elevated levels or increased sensitivity and is more commonly seen in multiple gestations or molar pregnancy [1]. It has also been reported to develop in pre-existing conditions like primary hypothyroidism and polycystic ovarian syndrome (PCOS) [1,2]. Thyroid-stimulating hormone (TSH) can act by specificity spillover phenomenon and lead to the development of HRL. It is important to suspect HRL clinically and on the basis of ultrasound and differentiate it from malignancy in order to avoid unwarranted interventions. We report a case of HRL developing as a result of undiagnosed primary hypothyroidism and regressing with the initiation of levothyroxine therapy.

## Case presentation

A 19-year-old Asian-Indian primigravida, in her 13th week of gestation, presented to the emergency medical services with complaints of progressive breathlessness, generalised abdominal distension and vomiting for three days. On further enquiry, she had a history of lethargy, malaise and constipation for the past six months. She complained of oligomenorrhoea for a year prior to pregnancy; however, she conceived spontaneously. She was not previously a known case of any medical illnesses. There was no history of hirsutism, acne or hair loss. There was no family history of hypothyroidism. On examination, she was afebrile, her pulse was 100 beats per minute and blood pressure was 100/60 mmHg. Her oxygen saturation was 96% on room air despite a modestly elevated respiratory rate of 22 breaths/min. A small firm goitre was palpable. There was no hirsutism or acanthosis nigricans. Abdominal examination revealed non-tender lower abdominal distension and a mass palpable till above the umbilicus that was disproportionate for the period of gestation. Systemic examination was normal. She was admitted to the hospital for further workup.

**Figure 1:**
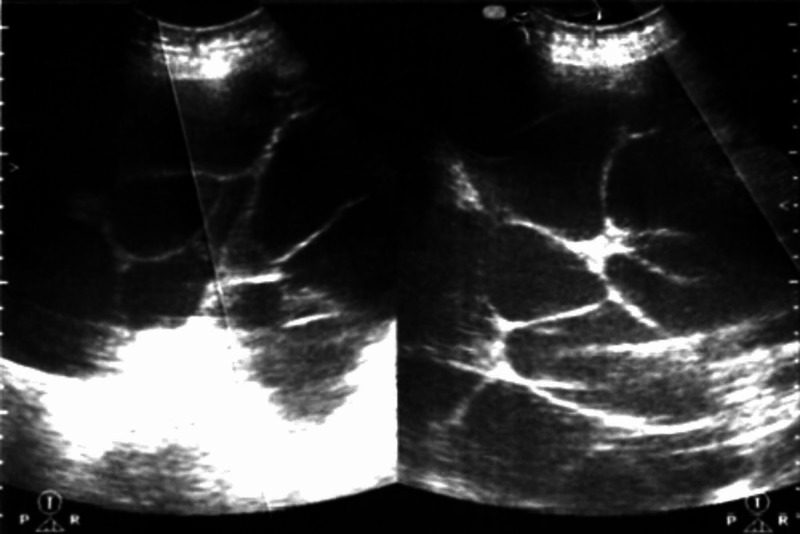
Ultrasound of ovaries showing bilateral cystic enlargement and a characteristic “spoke-wheel” pattern

Routine investigations including a complete blood profile, renal function tests and liver function tests were normal. An ultrasound of the abdomen and pelvis region demonstrated a single live intrauterine pregnancy of 13 weeks' gestation and cystic lesions in bilateral ovaries. Her right ovary measured 20.3 x 8.9 x 11.2 cm and left ovary measured 23 x 17.6 x 14.5 cm, both showing a “spoke-wheel” appearance (Figure 1).

Hormonal investigations suggested primary hypothyroidism with positive anti-thyroid peroxidase (anti-TPO) antibodies. Workup for ovarian cysts revealed elevated free and total testosterone levels and a normal cancer antigen-125 (CA-125) level. β-HCG was elevated, consistent with 13 weeks of gestation (Table 1).

**Table 1:** Summary of the hormonal profile of the patient TSH, thyroid-stimulating hormone; TPO, thyroid peroxidase; CA-125, cancer antigen-125; β-HCG, β-human chorionic gonadotropin

Investigation	Reference range	At presentation	Post-operatively	At six weeks' follow-up	Postpartum
Total T3	87 to 178 ng/dl	114.3			
Total T4	4.5 to 12.5 µg/dl	4.9			
Free T3	2.5 to 4.0 pg/ml	2.6			
Free T4	0.6 to 1.1 ng/dl	0.5			
TSH	0.35 to 5 µIU/ml	>150		2.2	2.4
Anti-TPO antibodies	<60 units/ml	>1300			
Total testosterone	0.2 to 0.8 ng/ml	5.8	4		0.1
Free testosterone	0.3 to 2 pg/ml	22.89			
CA-125	<35 IU/ml	10.38			
β-HCG (13th week of gestation)	13,300 to 254,000 mIU/ml	135,277			

When a patient presents with elevated testosterone levels and bilateral bulky ovaries in the fourth month of gestation, the possible differential diagnosis considered is discussed in Table 2.

**Table 2:** Summary of differential diagnosis when a pregnant patient presents with bilateral enlarged ovaries and hyperandrogenism Adapted from [1,3-8] OHSS, ovarian hyperstimulation syndrome; PCOS, polycystic ovarian syndrome; CA-125, cancer antigen-125

Characteristic	Ovarian tumour	Luteoma of pregnancy	OHSS	HRL/theca lutein cysts	Our patient
Laterality	Sertoli-Leydig tumours 5% bilateral, Krukenberg tumours 80% bilateral	47% bilateral	Typically bilateral	96% bilateral	Bilateral
Maternal androgen levels	Increased	Increased	Variable, may be increased as a result of the underlying cause of infertility	Increased	Increased
Maternal virilization	70%-100%	25%-35%	Rare, due to abrupt course	29.8%	Absent
Foetal virilization	Usually high, if mother virilized	High, if mother virilized	Rare	Rare (3.5%)	Absent
Important predisposing conditions	-	PCOS	Ovulation induction therapy	Gestational trophoblastic disease 80%-90%, primary hypothyroidism 16%	Primary hypothyroidism
Ultrasound	Presence of solid components, papillary projections or thick septations [1]	Commonly solid masses, occasionally with cystic areas, with single or multiple (50% cases) nodules [2,3]	Bilateral, often massively enlarged, multiple cysts of varying sizes with presence of ascites; may show a “spoke-wheel” pattern [4]	Multiple hypoechogenic cysts with absence of solid component; typical “spoke-wheel” pattern [4]	Bilaterally enlarged, cystic ovaries with a “spoke-wheel” pattern
Other features	Elevated CA-125	Third and fourth decades of life in multiparous women	Abrupt onset with dramatic presentation, luteal phase or early first trimester	Second to third trimester, indolent course	Young primigravida, fourth month of gestation, normal CA-125 level

Based on the history of a spontaneous pregnancy, ultrasonography findings, normal CA-125 and concurrent primary hypothyroidism, a diagnosis of HRL was concluded.

**Figure 2:**
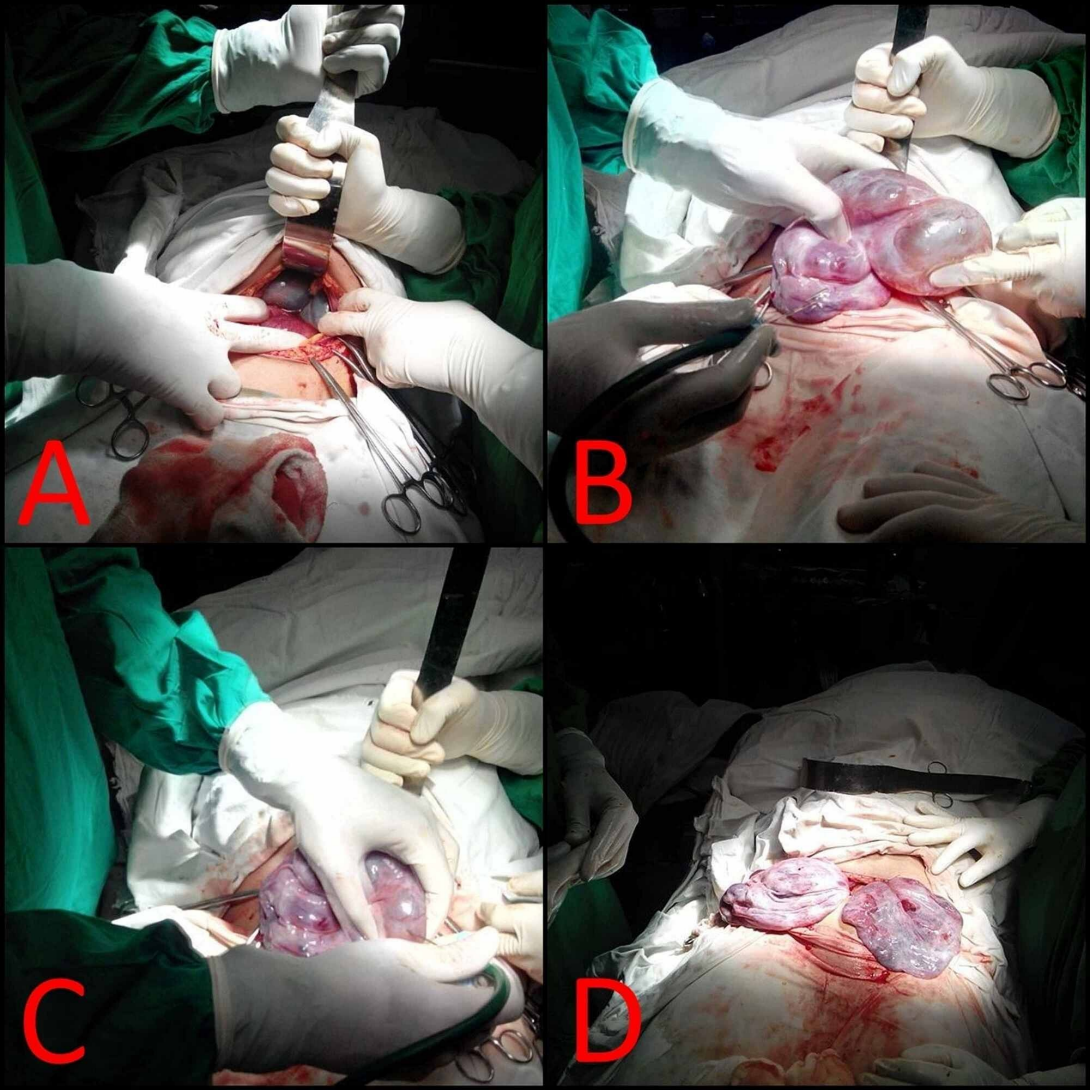
Exploratory laparotomy with cyst aspiration was performed on the third day of admission (A) Intraoperative bilateral enlarged multicystic ovaries. (B, C) Cyst aspiration being performed. (D) Collapse of ovaries following cyst aspiration.

The patient was initiated on levothyroxine replacement of 150 µg/day. However, on the third day of admission, her respiratory distress worsened for which she underwent emergency laparotomy. Intraoperatively, both ovaries were enlarged with a presence of multiple cysts of varying sizes between 4 and 7 cm. Cyst aspiration was performed and both ovaries collapsed intraoperatively, as shown in Figures 2A-2D.

**Figure 3:**
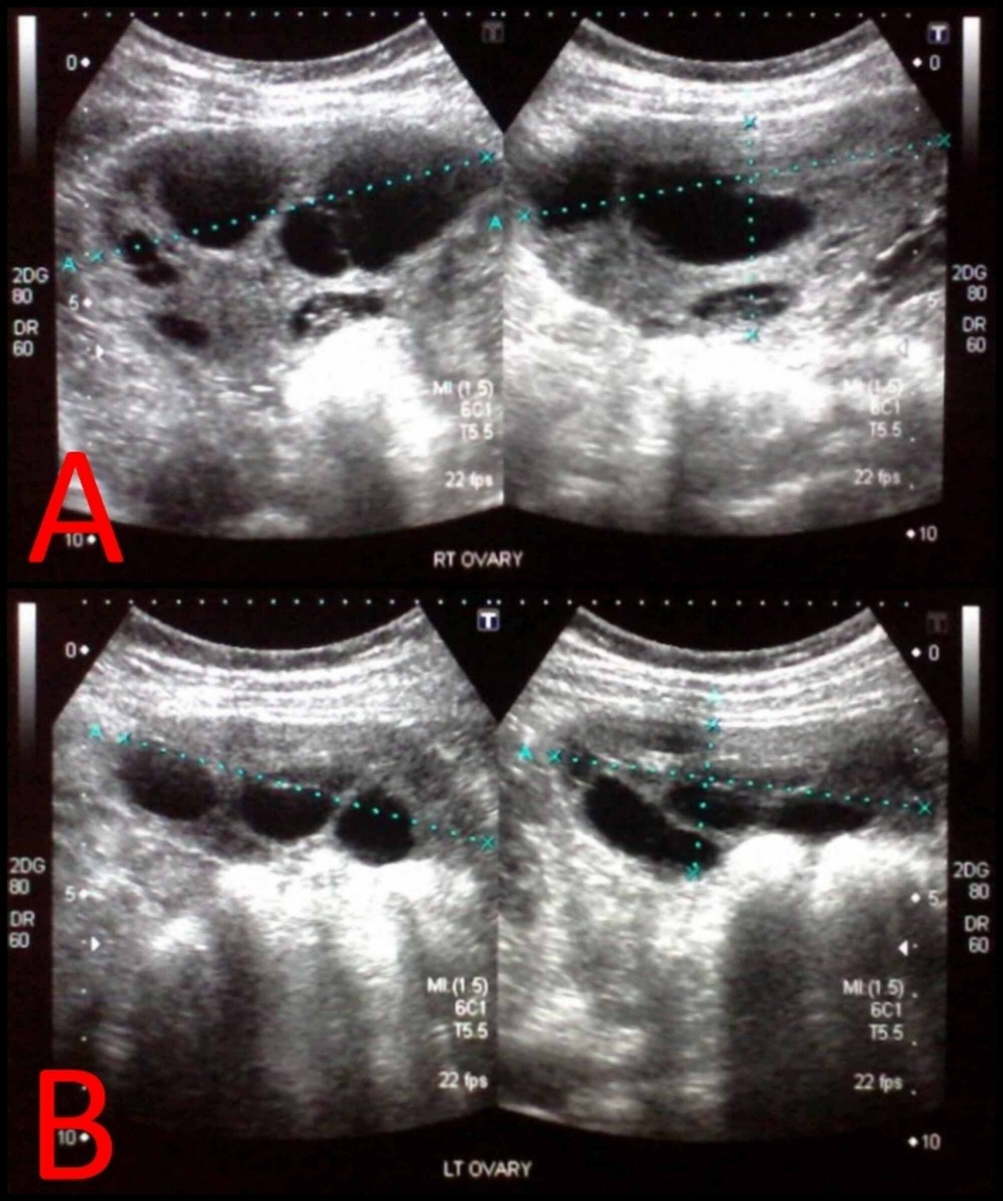
Post-operative ultrasound showing reduction in the size of (A) right and (B) left ovaries

One week after the initiation of levothyroxine, serum testosterone levels fell to 4 ng/ml. There was a decrease in the size of cysts bilaterally on follow-up ultrasound, with the right ovary measuring 8.9 x 8.4 x 4.4 cm (Figure 3A) and left ovary measuring 7.7 x 7.9 x 3.0 cm (Figure 3B). Six weeks after initiating levothyroxine therapy, TSH levels normalised to 2.2 µIU/ml. She was followed up monthly and TSH levels remained within the trimester-specific normal range. She delivered a healthy male child via a caesarean section at full term. Six weeks after delivery, serum testosterone was normal (0.1 ng/ml).

## Discussion

Hyperreactio luteinalis is a self-limiting benign condition that occurs during pregnancy and resolves spontaneously postpartum [9]. It is characterized by enlarged ovaries due to luteinized follicle cysts, as an extreme variant of theca lutein cysts. These cysts are believed to form in response to β-HCG over-stimulation, either as a result of elevated levels or increased sensitivity to normal levels of β-HCG [2,10]. Hence, the level of β-HCG required to cause HRL is extremely variable. Notably, in our patient, the β-HCG level was appropriate for her gestational age.

HRL consists mainly of multiloculated cystic lesions without internal mass and may often be mistaken for malignancy [11]. The cysts are predominantly filled with clear to blood-tinged fluid, although solid components may be present. Microscopically, they are composed of an inner layer of granulosa cells and an outer layer of theca interna cells showing markedly prominent luteinization [12].

It may be detected at any duration of gestation; however, the mean age of gestation is around 19.9 weeks [1]. Theca lutein cysts are often associated with molar pregnancies, with cysts prevailing in the tune of up to 50% of such cases [1,10]. However, as many as 60% to 80% of HRL cases described are singleton pregnancies and 67% are primiparous [1,9]. Our patient was a singleton primigravida in her second trimester, consistent with the described literature.

The risk factors for HRL are multiple gestations, Rh sensitization, twin to twin transfusion syndrome, hydrops fetalis, gestational trophoblastic neoplasia, molar pregnancy, choriocarcinoma, primary hypothyroidism, prior history of PCOS, gestational diabetes, ovulation induction with clomiphene and renal dysfunction (delaying the clearance of β-HCG) [13].

The structure of β-HCG and its many variants has been proposed as a contributing factor for the development of cysts and in turn for HRL. One particular variant lacking the C-terminal peptide of the β-subunit of the glycoprotein, almost identical to luteinizing hormone (LH), has been implicated to stimulate the ovary. The structural similarity between β-HCG, follicle-stimulating hormone (FSH), LH and TSH is responsible for the possible association of HRL with primary hypothyroidism, FSH-secreting adenoma, and PCOS [1,2,13,14]. Extremely elevated levels of TSH act via FSH receptors to cause follicular stimulation and via LH/β-HCG receptors to secrete testosterone from theca cells. It can also increase ovarian sensitivity to gonadotropins [2]. In our patient, unrecognized primary hypothyroidism with extremely high TSH levels, along with β-HCG, could have contributed to ovarian enlargement and hyperandrogenism. Treatment with levothyroxine led to the resolution of hyperandrogenism.

In the largest review till date, Malinowski et al. in 2015 reviewed 58 patients reported in the literature [1]. A brief comparison of the review with our patient is shown in Table 3.

**Table 3:** Comparison of patients reported in the literature with our patient PCOS, polycystic ovarian syndrome; CA-125, cancer antigen-125; β-HCG, β-human chorionic gonadotropin

Characteristic	Literature	Our patient
Maternal age	27.7 years (mean)	19 years
Gestational age	19.9 weeks (mean)	13 weeks
Pregnancy	81% Singleton	Singleton
	13.8% Twins	
	5.2% Triplets	
Gravidity	67% Primiparous	Primigravida
Conception	93.1% Spontaneous	Spontaneous
	6.9% Induced	
Virilization	29.8% Maternal	No virilization
	3.5% Foetal	
Comorbidities	6.9% PCOS	Hypothyroidism
	3.4% Hypothyroid	
	8.6% Hyperthyroid	
Investigations	84% Elevated testosterone	Elevated testosterone and β-HCG
	68.8% Elevated β-HCG	
Suspicion of malignancy	76.2%	Normal CA-125
Adverse events	25.9% Overall	Respiratory distress
Surgery	36.2% Overall	Aspiration of cyst

Clinically, patients usually present with a history of nausea and vomiting, consistent with elevated levels of β-HCG [15]. Patients may be asymptomatic and may present incidentally during a third trimester obstetric ultrasound or at caesarean section. Occasionally, patients may present with compressive symptoms such as abdominal discomfort, dyspnoea and abdominal pain. Maternal virilization due to hyperandrogenism is present in around 30% patients, most commonly in those presenting in the third trimester [1,13]. Increased levels of sex-hormone binding globulin during pregnancy may be responsible for the reduced frequency of clinical manifestations of hyperandrogenism [16]. Foetal virilization is even rarer occurring in 3.5% patients as HRL typically presents in second and third trimesters, when labioscrotal fusion has already occurred [1,16]. The placental aromatase activity also helps to protect the female foetus from virilization. There was no evidence of maternal or foetal virilization in our patient.

The possible complications include pre-eclampsia; hemolysis, elevated liver enzymes and low platelets (HELLP) syndrome; intra-uterine growth retardation; premature labour; ovarian torsion; dystocia; delayed lactation and foetal reproductive defects [13]. β-HCG has been linked with many regulatory functions in pregnancy including cytotrophoblast differentiation, angiogenesis and foetal growth and organogenesis. However, elevated β-HCG levels have been associated with adverse pregnancy outcomes, explaining the possible complications associated with HRL [17]. Fortunately, our patient did not have any of these obstetric complications, although respiratory distress did arise as a result of ovarian enlargement.

The diagnosis of HRL is usually established by a characteristic “spoke-wheel” appearance of the enlarged ovaries on ultrasound; however, a similar picture may be seen in ovarian hyperstimulation syndrome [5,18]. Magnetic resonance imaging may be performed, but serves no additional utility to an ultrasound.

Historically, surgical intervention was common due to difficulties in diagnosis and unnecessary oophorectomy was often performed due to suspicion of malignancy. However, with more advanced knowledge of radiological characteristics and natural course of the disease, a conservative approach is the rule rather than an exception. It usually takes around two months postpartum for the ovaries to return to their normal size [1]. Common indications, at present, for surgical intervention include an undue suspicion of malignancy or the presence of acute complications such as torsion, rupture of cysts or hemoperitoneum [19]. Ultrasound-guided percutaneous aspiration of cysts has been performed in the past as a measure to avoid surgery for torsion [20]. However, in our patient, due to significant respiratory distress, we performed emergency open laparotomy with aspiration of cysts.

## Conclusions

Primary hypothyroidism needs to be sought for in a pregnant patient presenting with enlarged cystic ovaries and elevated testosterone levels as it represents a treatable cause. The ovarian enlargement and resulting hyperandrogenism develops as a response to elevated TSH and regresses with levothyroxine replacement. It is imperative to recognize this condition clinically and by imaging and differentiate it from malignancy as conservative treatment is the management of choice and may avoid unwarranted oophorectomy.
